# Micro-nano aeration oxygenation and column P gradient variations enhance the soil microbial environment, thereby increasing corn yield

**DOI:** 10.3389/fpls.2025.1668095

**Published:** 2026-01-22

**Authors:** Jiayue Wang, Yupeng Zhao, Yanbo Fu, Zhiguo Wang, Wenlong Zhang, Jinquan Zhu

**Affiliations:** 1 College of Resources and Environment, Xinjiang Agricultural University, Urumqi, China; 2 Institute of Agricultural Resources and Environment, Academy of Agricultural Sciences of Xinjiang Uygur Autonomous Region, Urumqi, China; 3 College of Agriculture, Tarim University, Alar, China

**Keywords:** column P gradient, corn yield, enzyme activity, micro-nano aeration, soil microorganisms

## Abstract

In field cultivation, crop yields are frequently limited by soil compaction and low fertilizer use efficiency. Regulating the rhizosphere microbial community structure of crops may represent an effective mitigation strategy. This study investigated the effects of micro-nano aeration oxygenation (WP) and Column P gradient levels on the microbial environment of maize rhizosphere soil and their impact on maize yield. Results demonstrate that WP significantly increased maize yield and biomass: under the medium Column P gradient (PM), the yield in the WP treatment increased from 1104.93 kg/hm² to 1387.04 kg/hm² compared to the control treatment (CP), representing a 25.56% increase (p<0.05). Soil and plant analyses further showed that, under identical Column P gradients, WP promoted more efficient absorption and utilization of available phosphorus than CP. Furthermore, by increasing soil oxygen content and improving aeration, WP enhanced enzyme activity and microbial diversity in the rhizosphere soil. Collectively, these findings indicate that combining micro-nano aeration oxygenation with appropriate phosphorus application can effectively stimulate rhizosphere microbial activity, thereby promoting maize growth and nutrient use efficiency. This approach offers a theoretical basis for optimizing irrigation and fertilization strategies in maize production systems.

## 1 Introduction

Maize (Zea mays L.) plays a pivotal role in ensuring global food security and ranks among the most extensively cultivated crops worldwide (Henningsen et al., 2022). China is the second-largest maize producer globally, with approximately 36.8% of its total cultivated land (around 43.55 million hectares) dedicated to maize production. In 2020, Xinjiang, a key region for agricultural production and food security in China, had 36.8% of its cultivated land under maize cultivation, contributing an annual maize output of 9.28 million tons—accounting for 59% of the region’s total grain output and 3.65% of national maize output (Nbs, 2022; Zhu et al., 2024). Nevertheless, agricultural development in Xinjiang faces significant challenges, including soil salinization, inadequate fertility, and limited water resources (Li and Li, 2023), which negatively impact soil biodiversity, nutrient use efficiency, and maize productivity.

Phosphorus (P) is a critical factor for plant growth (Jin et al., 2023; Yang et al., 2024). However, soils in Xinjiang are predominantly alkaline, characterized by high phosphorus fixation rates (Khan F. et al., 2023) and low bioavailability (Adnan et al., 2020). This severely restricts maize phosphorus uptake, thereby limiting crop yield (Kazadi et al., 2022). Consequently, identifying efficient agricultural management practices to enhance phosphorus availability is essential for improving crop productivity (Li and Li, 2023).

In agricultural production, maintaining appropriate soil and water oxygen levels is vital for crop growth. Traditionally, chemical and biological oxygenation have been widely applied but exhibit limitations. Chemical oxygenation may cause significant fluctuations in soil pH, disrupting the soil microecological environment (Chauhan et al., 2024; Wang Q. et al., 2024; Uddin et al., 2025). For instance, chemical fertilizer application can induce soil acidification, reduce microbial abundance, disrupt soil aggregate structure, and decrease water-holding capacity and aeration (Uddin et al., 2025). Although biological aeration is environmentally friendly, its effective depth is often limited, making it difficult to alleviate hypoxic conditions in deep soil (Chauhan et al., 2024; Wang Q. et al., 2024). In contrast, micro-nano aeration oxygenation (WP) overcomes these limitations due to its smaller bubble diameter (Ye et al., 2023), enhanced soil penetration capacity, and environmentally friendly, non-polluting properties (DeBoer et al., 2024). Micro-nano aeration oxygenation improves water quality and increases soil oxygen concentration, thereby altering the composition and diversity of soil bacterial communities (Wang Q. et al., 2024), promoting the secretion of acid phosphatase and urease by aerobic bacteria, and accelerating organic phosphorus mineralization (Rawat et al., 2020; Bashir et al., 2024). It also accelerates the degradation of residual soil organic matter, providing carbon sources for microorganisms to further enhance metabolic activity (Bian et al., 2025). Organic acids secreted by actinomycetes and mycorrhizal fungi lower soil pH (Bian et al., 2024), promoting the dissolution of insoluble calcium phosphate and iron phosphate, reducing phosphorus fixation. Additionally, the micro-oxygen environment inhibits the activity of iron-reducing bacteria (Naorem et al., 2023), decreasing phosphorus adsorption and fixation by Fe²^+^ and avoiding deep phosphorus leaching loss (Sharifi et al., 2024). Meanwhile, micro-nano aeration oxygenation irrigation promotes aerobic respiration in plant roots, facilitating deeper root growth and enhancing root vitality and nutrient absorption capacity (Wang R. et al., 2023). This enhanced root growth not only promotes nutrient accumulation in plant tissues but also significantly increases crop yield and quality (Liu et al., 2019). Studies have shown that oxygenated water increases yields of tomatoes (Wang JW. et al., 2024), maize (Zhou et al., 2020), cotton (Abuarab et al., 2013), and cucumbers (Pendergast et al., 2013).After analysis and exploration, our team has gained a preliminary understanding of the impact of different Column P gradients on corn growth and the changes in soil microbial communities under micro-nano aeration conditions (Bian et al., 2024; Bian et al., 2025). This new study was conducted at the same location, using the same phosphorus application rate as Bian et al (Bian et al., 2025), and also incorporating the Column P gradient design for each phosphorus application rate. It also includes microbial network analysis to address the existing gaps in our understanding of phosphorus-oxygen interactions and possible underlying microbial mechanisms. To further innovatively investigate the regulatory effect of the interaction intensity of microbial networks on phosphorus availability, aiming to provide a more complete theoretical basis for the optimized application of micro-nano aeration technology.

This study used maize as the test crop and conducted field comparative experiments to systematically investigate the effects of micro-nano aeration oxygenation irrigation on soil physicochemical properties, enzyme activity, and microbial community structure, and to clarify their intrinsic relationships with maize growth and yield. The core scientific questions are as follows: (1) Investigate soil phosphorus utilization, accumulation, and plant phosphorus uptake under oxygenated irrigation to explore the role of oxygenated irrigation in maize phosphorus absorption and utilization; (2) Through studying soil microbial and enzyme activities, determine whether changes in soil microbial community activity under oxygenated irrigation are the core drivers of improved soil functions; (3) Examine the effects of oxygenated irrigation and Column P gradients on maize yield and biomass to clarify the relationships between oxygenated irrigation, maize yield, and Column P gradients; (4) By researching maize yield and soil physicochemical properties, explore whether the synergistic effect of oxygenated irrigation on the soil-crop system can stably enhance maize productivity. The results of this study will provide a new perspective for revealing the mechanism by which oxygenated irrigation improves soil health and offer practical guidance for the application of water-saving and efficiency-enhancing technologies in arid-region maize production systems.

## 2 Experimental design and methodological framework

### 2.1 Geographical characterization of the study area

**Figure 1:**
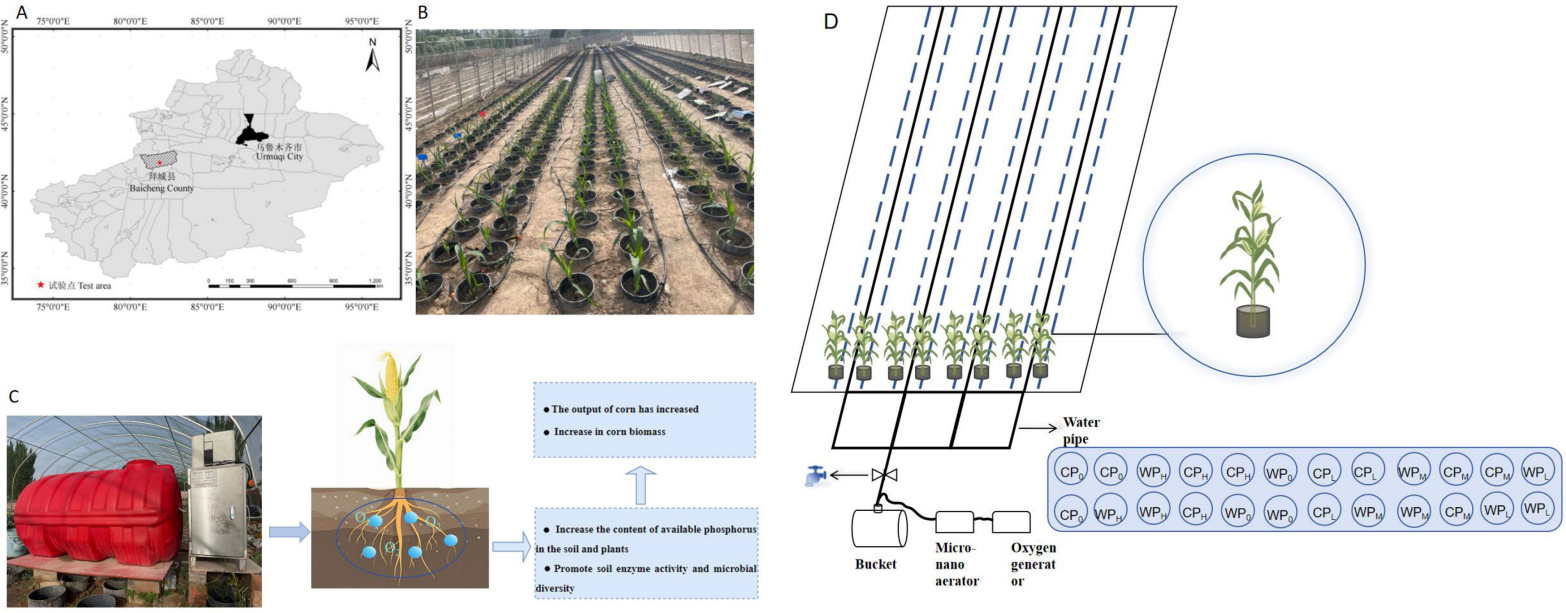
**(A)** The geographical location. **(B)** Real scene pictures of the experimental area. **(C)** Schematic diagram of the mechanism by which micro-nano aeration and oxygenation promote corn growth. **(D)** Experimental design of Column P gradient and simulation diagram of application scenarios.treatment abbreviations are defined in **Table 2**.

The field experiment was conducted during the 2023 growing season at the National Soil Quality Observation and Research Station of Aksu, located in the agricultural region of Baicheng County, Xinjiang (41°11’N, 80°15’E)(**Figure 1**). The experimental site is characterized by typical brown desert soil (Haplic Calcisols, FAO classification) with a sandy loam texture and moderate fertility. The region has a cold desert climate, exhibiting marked diurnal temperature variations, with the following mean annual climatic parameters: mean temperature 7.6 °C, maximum temperature 38.3 °C, minimum temperature -28.0 °C, frost-free period 148 days, annual sunshine duration of 2,789.7 hours, and annual precipitation of 171.1 mm. The station is equipped with comprehensive irrigation networks and standardized experimental plots, providing essential infrastructure for controlled experiments in maize cultivation research.

### 2.2 Experimental materials specification

Soil samples were collected from the National Soil Quality Observation and Experiment Station in Aksu (41°10’N, 80°14’E), a long-term experimental site under zero-fertilizer management since 2020. The brown desert soil had a sandy clay loam texture. Composite samples were collected from two distinct layers—the surface tillage horizon (0–20 cm) and the subsurface layer (20–90 cm)—using stainless steel augers. After air-drying at ambient temperature (25 °C) and sieving through a 2-mm mesh, the homogenized subsamples were stored in polyethylene containers until analysis (Bian et al., 2024; Bian et al., 2025). Basic physicochemical parameters, including bulk density, pH, and cation exchange capacity, are provided in **Table 1**.

**Table 1:** Physical and chemical properties of rhizosphere soil.

PH	Hydrolyzed nitrogen (mg/kg)	Organic matter (g/kg)	Available phosphorus (mg/kg)	Potassium (mg/kg)	Water-soluble salt (g/kg)	Carbon-nitrogen ratio (%)
8.12	107	27.2	14.7	409	3.1	16.1

The experimental maize variety was “Tianyu 303,” provided by Xinjiang Tianyu Co., Ltd. (Xinjiang, China). 15N-labeled urea (10.24% abundance) was used as the nitrogen fertilizer, purchased from Shanghai Chemical Research Institute (Shanghai, China). Phosphorus and potassium fertilizers were sourced from superphosphate (P_2_O_5_:45%) and potassium sulfate (K_2_O:50%), respectively. The micro-nano aeration oxygenation equipment used was a “B&W” micro-nano bubble generator (Honshu (Beijing) New Technology Promotion Co., Ltd., Beijing, China), which produces micro-nano bubble water. Its operating parameters were: working pressure, 0.015 MPa; and intake flow rate, 1.5 L·min^-^¹. The oxygen supply device was a YUyue YU300-type oxygen generator (manufactured by Jiangsu Yuyue Medical Equipment Co., Ltd., Jiangsu, China), with an oxygen flow rate range of 1–5 L·min^-^¹.

### 2.3 Experimental design

The soil column cultivation experiment was conducted using a randomized complete block design with a factorial arrangement(**Figure 1D**). Phosphorus (P_2_O_5_) application rates included four levels: P0: 0 kg ha^-^¹ (control), PL: 86 kg ha^-^¹, PM: 172 kg ha^-^¹, and PH: 258 kg ha^-^¹,Among these, PM denotes the recommended rate of phosphorus fertilizer application in local maize production, while PL and PH represent 50% reduction and 50% increase relative to the recommended rate, respectively. corresponding to dry soil mass-based application rates of 0, 0.028, 0.056, and 0.082 g P kg^-^¹ soil, respectively. Nitrogen fertilization (Urea-¹^5^N) was applied via flood irrigation in three split doses: 30% at sowing, 30% at the late vegetative stage, and 40% at the reproductive stage. Oxygenation treatments consisted of two levels: C: Control (ambient aeration) and W: Micro-nano aeration oxygenation (WP). These factors formed a 4×2 factorial design, resulting in eight experimental treatments, each replicated six times, with column positions randomized and maintained throughout the growth cycle. The specific meanings of each abbreviation in this article are shown in **Table 2**.

**Table 2:** The table listing the specific meanings of each treatment abbreviation in this study.

dummy suffix notation	P	P0	PL	PM	PH	C	W
concrete implication	Column P gradient	No phosphorus application,0 kg ha^-1^(control)	Local recommended amount-50%,86 kg ha^-1^	Local recommended quantity,172kg ha^-1^	Local recommendation +50%,258 kg ha^-1^	Conventional irrigation water	Micro-nano aeration oxygenated water

### 2.4 Experimental method

**Figure 2:**
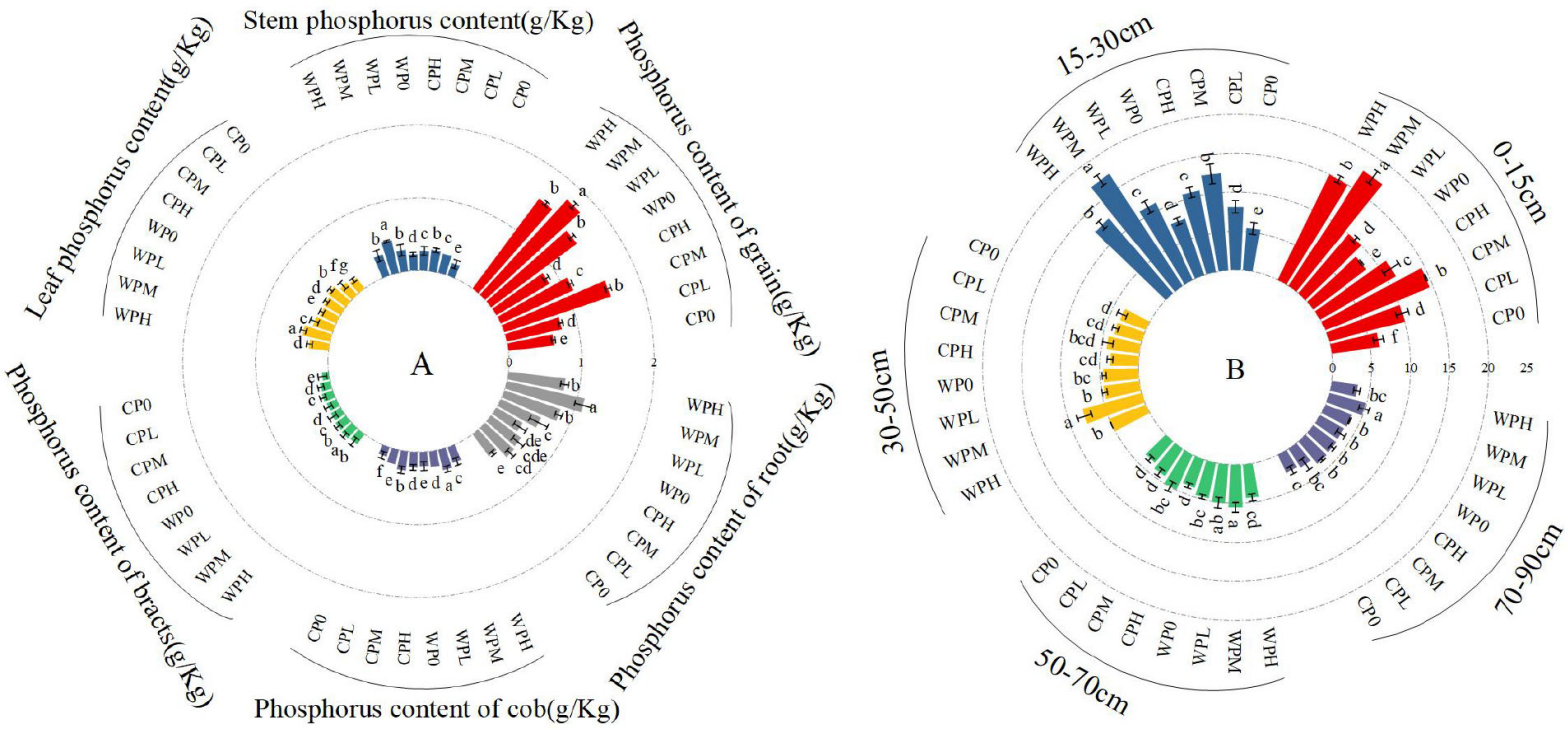
The effect of micro-nano aeration oxygenation on the available phosphorus content in soil and plants. In the figure, **(A)** represents the available phosphorus content of each part of the plant, and **(B)** represents the available phosphorus content of each layer of soil. Through one-way analysis of variance (ANOVA) and Duncan’s *post-hoc* test, The mean values of different treatments for the same corn variety indicated by different lowercase letters showed significant differences at the probability level of 0.05 (p < 0.05). The data are expressed as the mean ± standard deviation (SD) obtained from three repeated calculations.

This study employed the soil column cultivation approach. To minimize interference from external environmental factors, PVC pipes (inner diameter: 25 cm; height: 100 cm) were used and buried in the soil. The upper end was positioned 5 cm above ground level to prevent inflow of rainfall-induced surface runoff, while the lower end remained unsealed, maintaining direct contact with the natural soil. Each soil column was filled with 50 kg of dry soil in two layers: the 30–90 cm layer was collected from the 30–90 cm soil depth in the field, and the 0–30 cm layer was taken from the field plough layer (0–30 cm) (**Figure 2**). The 0–30 cm soil was thoroughly mixed with fertilizer before filling. After each filling step, the soil was watered and compacted to simulate natural field cultivation conditions.

Uniformly sized “Tianyu 303” maize seeds were selected. Sowing was performed manually via spot sowing on May 1, 2023, with a sowing depth of 2 cm and 4 seeds planted per soil column.

### 2.5 Experimental investigation

Soil sampling was conducted immediately after harvest (October 1, 2023) using stainless steel augers for stratified sampling from soil columns. Five depth intervals were sampled systematically: 0–15, 15–30, 30–50, 50–70, and 70–90 cm. Composite samples from each treatment were homogenized using the quartering method to ensure spatial representativeness. Post-collection protocols included: (i) immediate storage in sterile polyethylene bags; (ii) cryogenic preservation with dry ice during transport; and (iii) long-term cryopreservation at -80 °C to stabilize extracellular enzyme activities and microbial community structure. All post-processing procedures were performed under laminar flow hoods with triple alcohol sterilization between samples. These protocols minimized cross-contamination risks while preserving soil physicochemical integrity for subsequent metagenomic and enzymatic analyses.

### 2.6 Detection methods

The total phosphorus content of the plants was determined by the H2SO4 digestion - molybdenum-antimony ascorbate colorimetric method. The available phosphorus in the soil was determined by the molybdenum-antimony colorimetric method using air-dried soil that had passed through an 18-mesh sieve and was extracted with a 0.5 mol/L sodium bicarbonate solution (Sinoppharmaceutical Superior Grade Pure) (liquid-soil ratio 10:1).The total phosphorus content in the soil was determined by digestion with H2SO4-HClO4 and using the molybdenum-antimony colorimetric resistance method.

### 2.7 Determination of corn grain yield and its constituent factors

Six maize plants were selected from each treatment for indoor assessment. Grains were naturally air-dried to a moisture content of approximately 12.5%. Maize yield and its components were determined, including the number of cobs per plant, rows per cob, and grains per row. After threshing, total grain weight and 100-grain weight were measured.

### 2.8 Determination of enzyme activity

At the maturity stage, soil samples from the 0–15 cm and 15–30 cm layers were collected from six soil columns per treatment. Soil sucrase activity was determined using the 3,5-dinitrosalicylic acid (DNS) colorimetric method. Soil urease activity was assayed via the indophenol blue colorimetric method. Soil phosphatase activity was measured using the sodium p-nitrophenyl phosphate colorimetric method.

### 2.9 PCR amplification and high-throughput sequencing

Primers 338F(5’-ACTCCTACGGGAGGCAGCA-3’) and 806R(5’-GGACTACHVGGGTWTCTAAT-3’) were used for PCR amplification of the V3-V4 regions of 16S rRNA in rhizosphere soil bacteria; Primers ITS5F(5’-GGAAGTAAAAGTCGTAA CAAGG-3’) and ITS1R(5’-GCTGCGTTCT TCATCGATGC-3’) were used for PCR amplification of the ITS1 region of fungi, and the amplification system was (25µl): Reaction buffer (5×) 5µl, GC buffer (5×) 4 µl, 2.5 mm DNA TPS 2µl, forward and reverse primers (10 mm) 1µl each, DNA template 2µl, Q5DNA high-fidelity polymerase 0.25 µl and ddH2O 20µl. The amplification procedure is as follows: pre-denaturation at 95 °C for 2 minutes, denaturation at 98 °C for 15 seconds, annealing at 55 °C for 30 seconds, extension at 72 °C for 30 seconds, 25–30 cycles, and finally extension at 72 °C for 5 minutes (Chen et al., 2018). to quality control of the original sequencing sequence, Use FLASH (Magoč and Salzberg, 2023)software according to the degree of overlap between read spliced into tags; Then, these original labels were filtered using trimmi software (version 0.33) to obtain high-quality labels. Using UPARSE software (Edgar, 2013), according to 97% (Liu et al., 2019) the similarity of sequence of OTU clustering, Extract non-repetitive sequences from the optimized sequence and remove single sequences without repetitions. OTU clustering was performed on non-repetitive sequences (excluding single sequences) with a 97% similarity. Chimeras were removed during the clustering process to obtain representative OTU sequences. map all the optimized sequences to the OTU representative sequences and select the sequences with a similarity of more than 97% to the OTU representative sequences. After each sample of bacteria and fungi was divided, the analysis of microbial diversity and community composition was conducted.

## 3 Results

### 3.1 bubble oxygen-enriched irrigation modulating rhizosphere enzyme activation patterns and microbiome proliferation dynamics

Under micro-nano aeration oxygenation conditions, soil phosphorus transformation and utilization were promoted, effectively mitigating the adverse impacts of soil phosphorus loss on maize growth. Phosphorus distribution patterns across maize plant organs are illustrated in **Figure 2A**. Organ-specific phosphorus concentrations ranked in descending order: grains > roots > stems > leaves > cobs > bracts, with grains exhibiting the highest phosphorus accumulation and bracts the lowest. Oxygen-enriched phosphorus management (WPM) achieved optimal phosphorus concentrations in all examined organs. Comparative analysis revealed substantial enhancements under WPM treatment compared to non-oxygenated phosphorus treatments (CP0, CPL, CPM, CPH). Specifically, grain phosphorus concentrations increased by 180.6%, 128.7%, 14.9%, and 62.0% relative to CP0, CPL, CPM, and CPH treatments, respectively, while bracts showed respective increments of 103.7%, 32.6%, 18.8%, and 32.6%. These findings demonstrate that oxygen-coupled phosphorus application significantly improves phosphorus assimilation across maize organs, with WPM treatment exerting the most pronounced effect on phosphorus biofortification.

Soil available phosphorus displayed a vertical decreasing trend with increasing soil depth across all phosphorus treatments (**Figure 2B**). Comparative analysis indicated that moderate phosphorus application (PM) maintained the highest available phosphorus levels among the tested gradients, whereas high phosphorus treatment (PH) exhibited reduced accumulation efficiency. This confirms that optimized phosphorus application gradients enhance soil phosphorus bioavailability. Notably, the WPM treatment achieved superior phosphorus retention capacity, significantly outperforming other treatments in sustaining elevated available phosphorus concentrations throughout the soil profile (**Table 3**).

**Table 3:** Variance analysis of soil phosphorus content and plant phosphorus content by oxygenated irrigation, phosphorus application rate and their interaction factors.

Indicator		O	P	O*P
Soil phosphorus content	0-15	F=68.78,P<0.001	F=185.827,P<0.001	F=8.942,P<0.001
	15-30	F=79.86,P<0.001	F=111.743,P<0.001	F=3.468,P<0.05
	30-50	F=62.62,P<0.001	F=19.246,P<0.001	F=9.411,P<0.001
	50-70	F=15.34,P<0.001	F=6.759,P<0.05	F=0.366,NS
	70-90	F=10.912,P<0.05	F=6.999,P<0.05	F=2.054,NS
The phosphorus content of the plant	root	F=903.33,P<0.001	F=299.82,P<0.001	F=10.443,P<0.001
	stems	F=135.2,P<0.001	F=163.59,P<0.001	F=32.50,P<0.001
	leaf	F=320.64,P<0.001	F=330.56,P<0.001	F=27.12,P<0.001
	grain	F=232.58,P<0.001	F=220.06,P<0.001	F=18.43,P<0.001
	bracts	F=184.08,P<0.001	F=53.41,P<0.001	F=7.861,P<0.005
	ear axis	F=136.13,P<0.001	F=356.13,P<0.001	F=26.13,P<0.001

**In the table:**O represents oxygenated irrigation and P represents the amount of phosphorus application,The same below.

### 3.2 Impacts of micro-nano oxygen-enriched irrigation regimes on soil enzymatic dynamics and microbial community abundance

**Figure 3:**
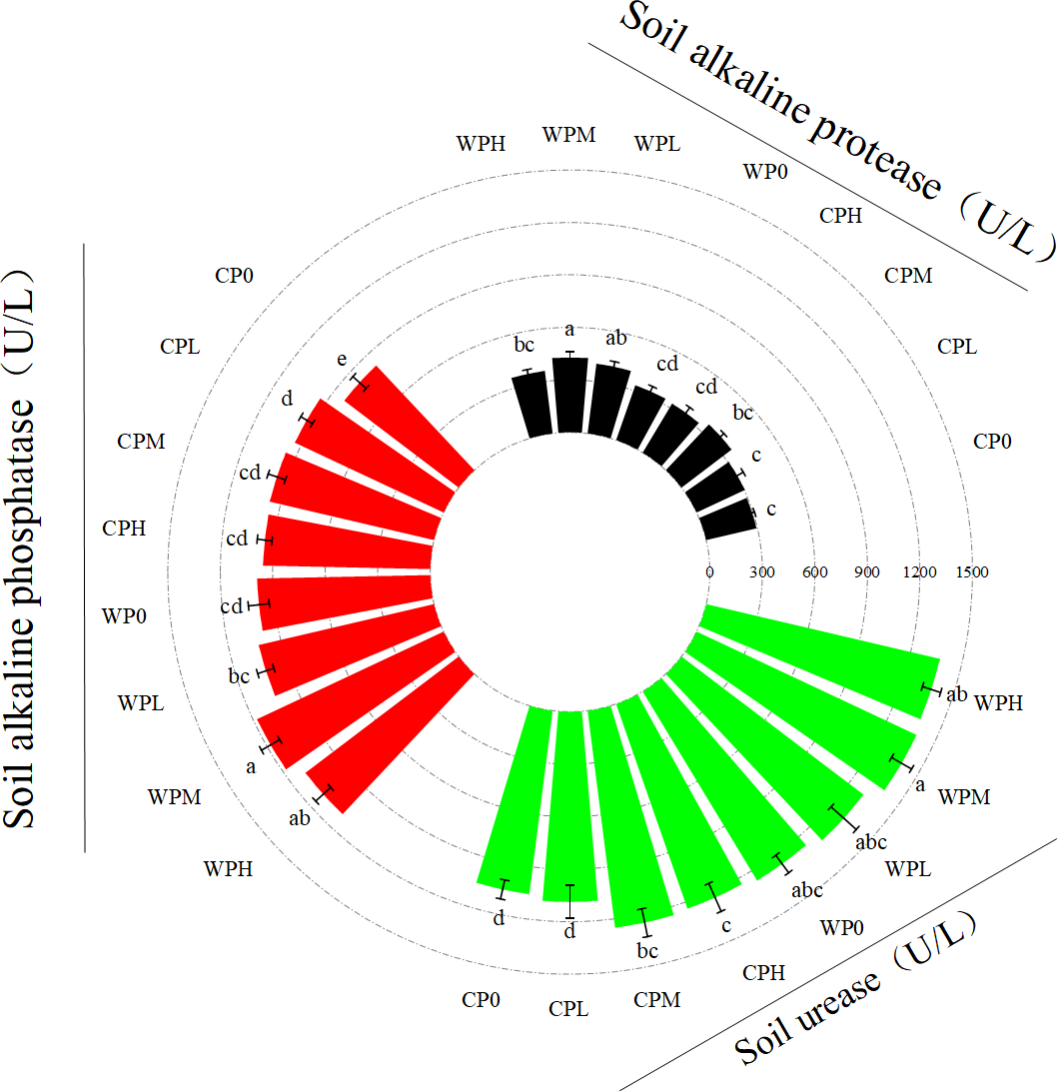
Effects of micro-nano aeration oxygenation on the activities of soil alkaline protease, soil alkaline phosphatase and soil urease. Through one-way analysis of variance (ANOVA) and Duncan’s *post-hoc* test, The mean values of different treatments for the same corn variety indicated by different lowercase letters showed significant differences at the probability level of 0.05 (p < 0.05). The data are expressed as the mean ± standard deviation (SD) obtained from three repeated calculations.

Micro-nano aeration oxygenation irrigation (WP) significantly enhances the activities of soil alkaline protease, alkaline phosphatase, and urease, while also exerting a stimulatory effect on soil microbial activity (**Figure 3**). The activities of alkaline protease, alkaline phosphatase, and urease varied significantly with irrigation method (P < 0.001). However, the interaction between irrigation method and Column P gradient was not statistically significant. Alkaline protease activity was significantly correlated with Column P gradient (P < 0.05), whereas alkaline phosphatase and urease activities showed stronger correlations (P < 0.001 and P < 0.01, respectively) (**Table 4**). Under oxygenation treatment, alkaline phosphatase activity increased with Column P gradient, peaking at the medium phosphorus level (PM). Specifically, the oxygenated medium phosphorus treatment (WPM) increased alkaline phosphatase activity by 43.56%, 25.42%, 22.25%, and 23.30% compared to non-oxygenated treatments (CP0, CPL, CPM, CPH), respectively. These results indicate that oxygenation combined with phosphorus application effectively enhances soil enzyme activity.

**Table 4:** Variance analysis of enzyme activity and microbial activity by oxygenated irrigation, phosphorus application rate and their interaction factors.

Indicator		O	P	O*P
Enzyme activity	Soil alkaline protease	23.554,p<0.001	7.476,p<0.05	1.511,NS
	Soil alkaline phosphatase	53.161,p<0.001	11.223,p<0.001	1.503,NS
	Soil urease	31.206,p<0.001	6.367,p<0.05	0.513,NS
Microorganism	Fungus chao1	9.059,p<0.01	4.162,NS	0.147,NS
	Fungal ACE	18.182,p<0.001	5.996,p<0.05	0.437,NS
	Bacteria chao1	14.007,p< 0.05	5.222,p<0.05	0.775,NS
	Bacteria ACE	11.064,p<0.05	4.137,NS	0.709,NS

**Figure 4:**
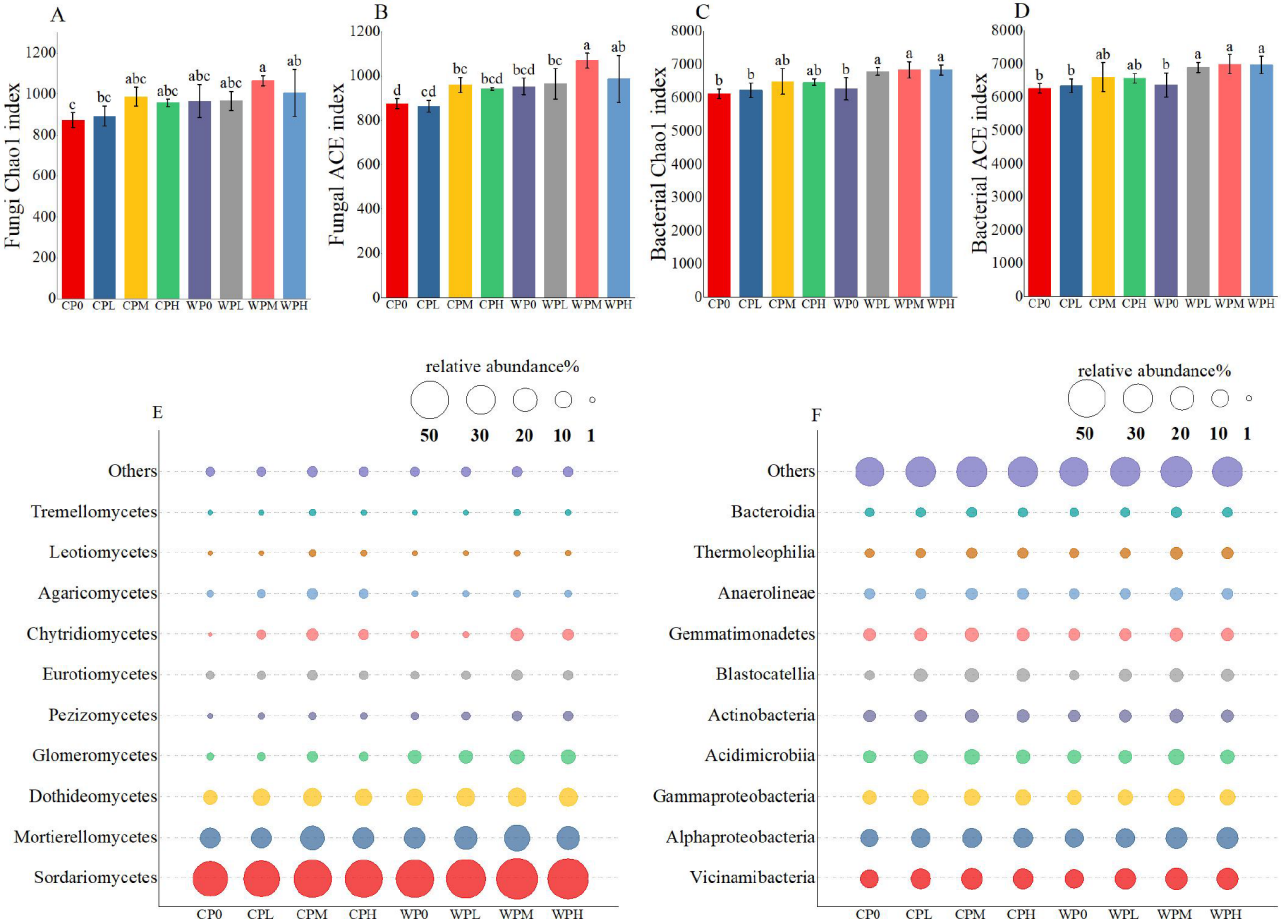
The effect of micro-nano aeration oxygenation on microbial abundance values and richness. **(A, B)** represents the fungal abundance index, and **(C, D)** represents the bacterial abundance index, **(E)** represents fungal richness and **(F)** represents bacterial richness. Through one-way analysis of variance (ANOVA) and Duncan’s *post-hoc* test, The mean values of different treatments for the same corn variety indicated by different lowercase letters showed significant differences at the probability level of 0.05 (p < 0.05). The data are expressed as the mean ± standard deviation (SD) obtained from three repeated calculations.

Analysis of bacterial and fungal richness indices (ACE and Chao1) revealed that oxygenation treatment significantly increased richness compared to conventional irrigation (**Figure 4A-D**). With increasing phosphorus application, richness indices increased in both conventional and oxygenation treatments. Notably, richness indices in oxygenated phosphorus treatments (WPL, WPM, WPH) were significantly higher than in the non-oxygenated, phosphorus-free treatment (WP0). Among these, WPM showed the highest richness. Specifically, fungal Chao1 richness in WPM was 22.1%, 19.3%, 7.83%, and 11.4% higher than in CP0, CPL, CPM, and CPH, respectively. For ACE, WPM was 22.2%, 23.8%, 11.4%, and 13.5% higher than CP0, CPL, CPM, and CPH, respectively. These results indicate that oxygenated phosphorus application significantly enhances fungal and bacterial richness, with WPM having the most pronounced effect.

To visualize microbial community composition, the top 10 most abundant taxa were selected for display (**Figure 4E, F**), with remaining taxa grouped as “Others.” At the phylum level, Vicinamibacteria, Alphaproteobacteria, and Gammaproteobacteria were the dominant bacterial taxa, while Sordariomycetes, Mortierellomycetes, and Dothideomycetes were the dominant fungal taxa. Microbial abundance in both non-oxygenated (C) and oxygenated (W) groups increased with Column P gradient, peaking at medium phosphorus (PM) and slightly decreasing at high phosphorus (PH). This indicates that under oxygenation, optimal phosphorus application effectively enhances microbial richness and diversity.

### 3.3 Oxygen-nanobubble irrigation regimes modulating soil enzymatic cascades and microbial consortia dynamics toward optimized Zea mays yield and biomass accumulation

**Figure 5:**
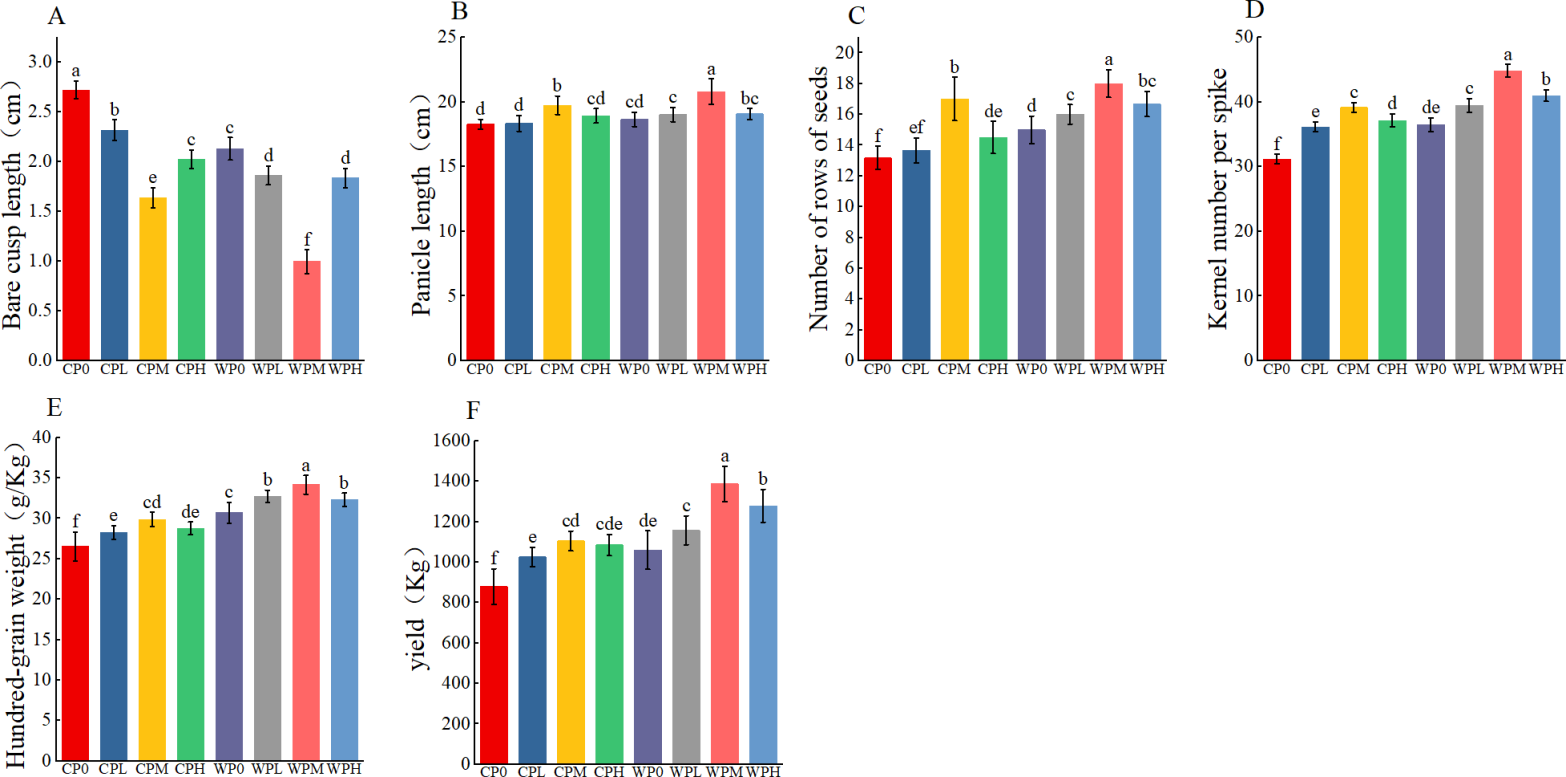
The influence of micro-nano aeration oxygenation irrigation on corn yield factors. **(A–F)** represent the bald tip length, ear length, number of grainrows, number of grains per ear, weight per 100 grains and yield of corn respectively. Through one-way analysis of variance (ANOVA) and Duncan’s *post-hoc* test, The mean values of different treatments for the same corn variety indicated by different lowercase letters showed significant differences at the probability level of 0.05 (p < 0.05). The data are expressed as the mean ± standard deviation (SD) obtained from three repeated calculations.

Micro-nano aeration oxygenation (WP) positively impacts maize yield and its yield components (**Figure 5F**). Oxygenated irrigation combined with phosphorus application significantly affected maize yield and 100-grain weight (p<0.001) (**Table 5**). Across all Column P gradients, yield and 100-grain weight under oxygenated conditions were significantly higher than those under non-oxygenated treatments (p<0.05). Under both oxygenated and non-oxygenated conditions, maize yield increased initially and then decreased with increasing phosphorus levels, peaking at the medium Column P gradient (PM). Specifically, the yield in the oxygenated medium phosphorus treatment (WPM) was 57.7%, 35.3%, 25.6%, and 27.9% higher than those in non-oxygenated treatments (CP0, CPL, CPM, and CPH), respectively. Phosphorus application significantly increased maize yield, with maximum yields observed at the medium phosphorus level (PM): 1,104.93 kg/hm² for non-oxygenated medium phosphorus (CPM) and 1,387.04 kg/hm² for oxygenated medium phosphorus (WPM).

**Table 5:** Variance analysis of oxygenated irrigation, phosphorus application rate and their interaction factors on maize yield and biomass.

Indicator		O	P	O*P
Corn yield	The weight of a hundred grains	170.04,p<0.001	19.18,p<0.001	0.40,NS
	Grain yield	85.67,p<0.001	31.7,p<0.001	2.16,NS
Biomass	Root trunk weight	15.71,p<0.001	1.48,NS	0.15,NS
	Stem weight	27.12,p<0.001	0.21,NS	0.13,NS
	Leaf dry weight	6.28,p<0.05	1.29,NS	1.20,NS
	Dry weight of the ear axis	14.36,p<0.001	0.86,NS	0.13,NS
	Dry weight of bracts	16.67,p<0.001	0.76,NS	0.37,NS
	Dry weight of grains	21.07,p<0.001	0.22,NS	0.36,NS

Dry matter accumulation in maize organs under different treatments is shown in **Figure 5**. Oxygenated irrigation significantly increased the dry matter content of all maize organs (p<0.05). Under both oxygenated and non-oxygenated conditions, dry matter accumulation in each organ increased initially and then decreased with increasing phosphorus levels, reaching maximum values at the medium Column P gradient (PM). Notably, the grain dry weight in the WPM treatment was 56.5%, 33.9%, 24.5%, and 26.9% higher than those in CP0, CPL, CPM, and CPH, respectively. The combination of oxygenated irrigation and optimal phosphorus application effectively improved overall dry matter accumulation in maize.

### 3.4 Correlation analysis of microorganisms and corn yield under micro-nano aeration and oxygenation irrigation conditions

**Figure 6:**
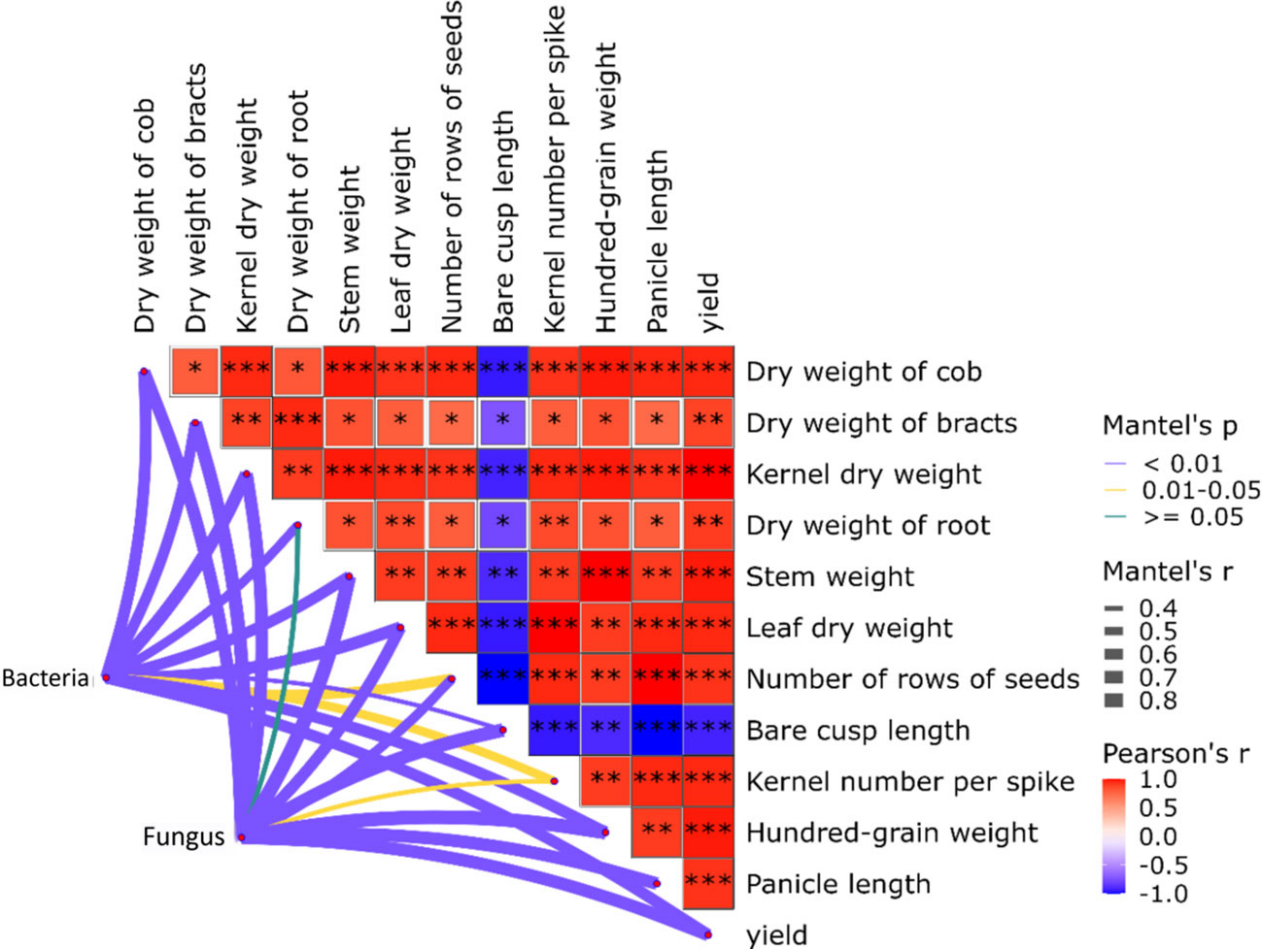
Correlation between microorganisms and corn yield factors.Red indicates a positive correlation and blue indicates a negative correlation.**, *** respectively represent correlations at 0.01 and 0.001.

To further explore relationships between microbial abundance, yield, and related indicators, correlation analysis was performed to examine these variables (**Figure 6**). For correlations between yield components and biomass indicators, bare tip length showed negative correlations with all measured indicators, with the strongest negative correlations observed for spike axis dry weight, grain dry weight, leaf dry weight, grain row number, grains per spike, spike length, and yield (p<0.05). Yield was significantly positively correlated with all other measured indicators, indicating that favorable maize growth status is critical for yield improvement.

In correlation analyses between bacterial/fungal species richness and maize yield components, bacterial and fungal richness exerted a highly significant positive effect on maize yield (p<0.01), suggesting that a favorable microbial environment promotes maize yield. Notably, bacterial richness was negatively correlated with root dry weight, though this relationship was not statistically significant (p>0.05), indicating an independent association between root dry weight and bacterial richness in this study. Collectively, these results demonstrate that microbial richness significantly influences maize yield and biomass.

Heat map analysis revealed that maize yield and plant biomass are interdependent and mutually reinforcing. Network graph analysis further indicated that microbial richness exhibits strong correlations with maize yield, with consistent trends observed. Thus, high microbial community richness is an important factor promoting maize yield.

## 4 Discussion

### 4.1 Micro-nano bubble oxygenation irrigation improves the absorption of phosphorus

The movement of phosphorus in soil is primarily governed by convection and adsorption mechanisms, and its efficiency is influenced by soil microorganisms (Stackebrandt and Note, 1994) and enzyme activity (Guo et al., 2023)Our team’s previous research has demonstrated that irrigation methods and Column P gradients significantly affect soil enzyme activity, thereby influencing phosphorus conversion efficiency (Bian et al., 2024; Bian et al., 2025). For instance, micro-nano aeration oxygenation (WP) significantly enhanced the activities of alkaline phosphatase, alkaline protease, and urease in the 0–15 cm soil layer. This indicates that WP improves the soil aerobic microbial environment, promoting microbial proliferation (Zhao et al., 2023; Zhou L. et al., 2023). Increased aerobic microbial activity accelerates organic matter decomposition and nutrient release (Wang J. et al., 2023; Zhou Q. et al., 2023), thereby facilitating the conversion of organic phosphorus to inorganic phosphorus and improving soil quality (Zhou et al., 2022; Zhang et al., 2023). This process supports normal crop growth and enhances phosphorus absorption efficiency. Our findings are consistent with previous studies and further confirm that under water scarcity and low phosphorus conditions, WP treatment remains effective in promoting plant phosphorus uptake and utilization (Liu et al., 2023). Additionally, our research highlights that different phosphorus application strategies significantly influence soil phosphorus mobility and availability. Excessive phosphorus application may lead to phosphorus accumulation and fixation (Urlić et al., 2023), reducing its utilization efficiency (Chen et al., 2025). In contrast, moderate phosphorus application combined with micro-nano bubble drip irrigation can prevent excessive phosphorus accumulation, maintain high phosphorus availability, and ensure efficient plant phosphorus absorption and utilization (Khan A. et al., 2023). These results corroborate our findings and underscore the importance of optimizing phosphorus application rates and irrigation methods to enhance soil phosphorus efficiency and crop yields.

### 4.2 Micro-nano bubble oxygenation irrigation promotes microbial abundance

Compared with the untreated control group (CP0), various irrigation treatments induced significant differences in bacterial abundance. Bacterial abundance increased markedly with increasing Column P gradient; notably, in the oxygenated medium phosphorus treatment (WPM), bacterial abundance was approximately 22.15% higher than in CP0 (Han et al., 2022). demonstrated that micro-nano aeration oxygenation (WP) significantly enhanced the richness and diversity of soil microorganisms while altering their community structure. By increasing soil oxygen content via micro-nano aeration intervention, the proliferation of oxygen-adapted microbial species was promoted (Niu et al., 2016; Sun et al., 2018), consequently inducing shifts in microbial community composition (Zhou et al., 2019). In this study, all top 10 most abundant fungal taxa were aerobic. Among bacteria, all dominant taxa were aerobic except for the anaerobic groups Blastocatellia, Anaerolineae, and Bacteroidia. Notably, these three anaerobic groups ranked 6th, 8th, and 9th, respectively, among the top 10 most abundant taxa. Correspondingly, under micro-nano aeration oxygenation (WP), the abundance of these three anaerobic groups was lower than in non-oxygenated treatments, consistent with (Chen et al., 2023). However, some studies have reported that micro-nano bubble oxygenated water application in tomato experiments significantly reduced rhizosphere microbial diversity. These discrepancies may be due to differences in oxygen content and require further investigation.

### 4.3 Micro-nano bubble oxygenation irrigation increases corn yield

This study investigated the comparative effects of micro-nano aeration oxygenation treatment (WP) and conventional treatment (CP) on maize yield. Micro-nano aeration oxygenation irrigation is widely recognized for enhancing crop yield by improving the soil root zone environment and promoting crop water and fertilizer uptake (Du et al., 2024). Under identical Column P gradients, micro-nano bubble oxygenation treatment significantly increased maize yield. Phosphorus is a critical resource for crop production (Ouyang et al., 2023). In this study, WP treatment significantly increased maize 100-grain weight, grain yield, and biomass. Specifically, under the medium phosphorus (PM) treatment with WP (WPM), the positive impact of Column P gradient on maize yield and biomass increased progressively with phosphorus levels, whereas the high phosphorus treatment exhibited diminishing returns. This aligns with previous findings (Amanullah and Khattak, 2009; Erel et al., 2013; Jorfi et al., 2022; Lopes Sobrinho et al., 2024). Phosphorus application significantly enhances maize grain weight and yield; however, exceeding a critical phosphorus application threshold reduces grain number, grain weight, and individual plant yield. This may result from excessive phosphorus fertilizer inhibiting maize uptake of other essential elements (Erel et al., 2016). Additionally, residual soil phosphorus can bind with minerals such as calcium and magnesium, which may exacerbate soil compaction. This is also the focus of our future research, as we aim to increase or overcome the phosphorus application threshold for maize.

#### 4.3.1 Benefits of micro-nano bubble aeration irrigation for maize cultivation in Baicheng County

Baicheng County has a temperate continental arid climate. The annual precipitation is low (171.13mm), the temperature fluctuates greatly (from a maximum of 38.3 °C to a minimum of -28 °C), the frost-free period is short (133–163 days), and the annual sunshine duration is abundant (2,789.7 hours). The prediction result of the optimal sowing window period in Baicheng County based on the Hybrid-Maize model is between April 25th and May 5th (Shen et al., 2020). Annual precipitation is expected to decrease by 8–12% by 2050, while potential evapotranspiration rises by 10–15% due to a 1.5–2.0 °C increase in average summer temperatures (He et al., 2024). This will exacerbate water scarcity, the most pressing constraint for local maize production (Meng et al., 2016). Summer heatwaves (daily maximum >35 °C) may triple in frequency, and short-duration heavy rainfall events (≥50mm/day) could increase by 15–20% (Rahimi-Moghaddam et al., 2018). These fluctuations will disrupt soil moisture balance—alternating between drought and waterlogging—and amplify root hypoxia under high temperatures (Singh et al., 2022; Hou et al., 2024). These predictions pose a serious threat to China’s corn industry: The corn output in northwest China accounts for approximately 30% of the national corn output, and regional yield declines could destabilize domestic supply (critical for food, feed, and bioenergy needs) amid rising global demand (Bi et al., 2019; Xing et al., 2025).Micro-nano bubble aeration irrigation technology, by optimizing the root-zone microenvironment, offers a promising solution to these constraints. In Baicheng County, the application of this technology has demonstrated multiple agronomic benefits for corn production. During summer months, extreme temperatures reduce soil oxygen solubility, leading to root hypoxia and impaired physiological functions (Striker et al., 2025). Micro-nano bubble aeration irrigation mitigates heat stress by enhancing soil dissolved oxygen levels (Abd Elhady et al., 2021). As detailed in Section 4.2, this technique promotes the proliferation of aerobic microorganisms (Sun et al., 2018; Chen et al., 2025) while suppressing anaerobic populations (Chen et al., 2023). This shift in microbial community structure enhances the activity of key soil enzymes—alkaline phosphatase, protease, and urease—which play vital roles in nutrient cycling and root health (Huang et al., 2025). Furthermore, improved root zone oxygenation reduces the accumulation of toxic metabolic byproducts (Prasad and Raghuwanshi, 2022), thereby maintaining root viability and photosynthetic efficiency under high-temperature conditions (as evidenced by an 11.81% increase in SPAD values observed in this study). The arid climate also limits the decomposition of organic matter, resulting in low bioavailability of soil nutrients, particularly phosphorus. Micro-nano bubble aeration irrigation improves nutrient utilization efficiency through enhanced microbial and enzymatic activities. As highlighted in Section 4.1, the technology increased alkaline phosphatase activity by 15.4%, accelerating the mineralization of organic phosphorus into plant-available inorganic forms. Coupled with a 30% improvement in phosphorus uptake efficiency, this reduces the need for excessive phosphorus fertilization—practices that often lead to nutrient fixation and environmental accumulation (Urlić et al., 2023; Chen et al., 2025). The integration of optimized phosphorus application with micro-nano bubble aeration irrigation sustains high phosphorus use efficiency, ensures adequate nutrient supply, and minimizes fertilizer waste. For local farmers in Baicheng County, this translates into reduced input costs and lower environmental risks. Given the short frost-free period (133–163 days), corn must complete its life cycle rapidly to avoid late-season frost damage. Micro-nano bubble aeration irrigation supports early growth and development by improving water and nutrient uptake. As shown in Section 4.3, this approach significantly increased biomass accumulation and grain yield (with the WPM treatment yielding 25.56% more than the CP0 control). The technology accelerates the transition from vegetative to reproductive growth, enabling earlier maturity and maximizing yield potential within the constraints of the local climate. Future research should focus on refining application protocols—such as timing and frequency—of micro-nano bubble aeration irrigation to further enhance its effectiveness under the specific agro-climatic conditions of Baicheng County.

### 4.4 Future research and development plans for micro-nano aeration oxygenation technology

The current study provides preliminary evidence for the synergistic benefits of micro-nano aeration oxygenation and optimized phosphorus management on maize yield and soil microbial health in Xinjiang’s arid agricultural system. However, its scope is limited to small-scale soil column experiments and a single crop type, necessitating further research to validate scalability, adaptability, and long-term sustainability across diverse agricultural contexts. Our team proposes the following future plans:

#### 4.4.1 Nationwide large-scale pilot testing

Over the next two years, we will implement a nationwide pilot program covering major agricultural ecological zones in China, including the Northeast Black Soil Region, North China Plain, Southern Red Soil Region, and Northwest Arid Region. These pilots will target diverse soil types (saline-alkali, acidic, sandy) and climatic conditions to evaluate WP’s regional adaptability. We will collect data on crop yield, nutrient use efficiency, and soil microbial dynamics to establish evidence-based guidelines for large-scale application and policy support.

#### 4.4.2 Expand to diversified planting systems

In addition to corn, we will also expand the application scope of micro-nano aeration and oxygenation to major crops (wheat, rice), horticultural crops (tomatoes, cucumbers), and economic crops (cotton, fruit trees). In addition, we will evaluate its performance in intercropping, crop rotation and organic farming systems to verify the universality of different planting patterns and the improvement of productivity.

#### 4.4.3 Promoting sustainable phosphorus use

Building on the optimal Column P gradient (PM) findings, future research will focus on:

Reducing Fertilizer Input: Quantifying WP’s potential to cut phosphorus application rates by enhancing phosphorus availability and uptake efficiency, minimizing fixation and environmental losses. Microbial Synergy: Combining WP with phosphate-solubilizing bacteria or mycorrhizal fungi to further improve phosphorus utilization and reduce chemical fertilizer reliance. Long-Term Soil Health: Evaluating WP’s long-term impact on soil phosphorus cycling and microbial stability to ensure sustainable agricultural production.

These plans will advance theoretical understanding of WP technology and provide practical solutions for improving crop productivity, nutrient efficiency, and soil health in China’s diverse agricultural systems.

## 5 Conclusion

Micro-nano aeration irrigation has been shown to improve the soil microbial environment, enhance phosphorus fertilizer utilization efficiency, and increase corn yield. The activities of key soil enzymes—alkaline phosphatase, alkaline protease, and urease—are significantly elevated, accompanied by an increased abundance of microbial communities and higher levels of available phosphorus in both soil and plants. This study preliminarily investigates the interaction between phosphorus and oxygen, as well as the potential regulatory role of soil microorganisms in phosphorus uptake and utilization by corn. These improvements collectively contribute to enhanced phosphorus use efficiency and higher crop productivity.

## Data Availability

The raw data supporting the conclusions of this article will be made available by the authors, without undue reservation.
