# Correction: Micro-nano aeration oxygenation and column P gradient variations enhance the soil microbial environment, thereby increasing corn yield

**DOI:** 10.3389/fpls.2026.1799373

**Published:** 2026-03-18

**Authors:** Jiayue Wang, Yupeng Zhao, Yanbo Fu, Zhiguo Wang, Wenlong Zhang, Jinquan Zhu

**Affiliations:** 1 College of Resources and Environment, Xinjiang Agricultural University, Urumqi, China; 2 Institute of Agricultural Resources and Environment, Academy of Agricultural Sciences of Xinjiang Uygur Autonomous Region, Urumqi, China; 3 College of Agriculture, Tarim University, Alar, China

**Keywords:** column P gradient, corn yield, enzyme activity, micro-nano aeration, soil microorganisms

There was a mistake in **Table 1** as published. The column “Organic matter (g/kg)” was duplicated. The corrected **Table 1** appears below.

**Table 1:** Physical and chemical properties of rhizosphere soil.

PH	Hydrolyzed nitrogen (mg/kg)	Organic matter (g/kg)	Available phosphorus (mg/kg)	Potassium (mg/kg)	Water-soluble salt (g/kg)	Carbon-nitrogen ratio (%)
8.12	107	27.2	14.7	409	3.1	16.1

The original version of this article has been updated.

